# Thermodynamic Analysis of Myelin Basic Protein Adsorbed on Liquid Crystalline Dioleoylphosphatidylcholine Monolayer

**DOI:** 10.1155/2019/8175413

**Published:** 2019-11-04

**Authors:** Zhang Lei, Sun Runguang, Hao Changchun, Yang Huihui, Hu Chengxi

**Affiliations:** ^1^Department of Experimental Teaching Center for Optoelectronic Science and Information Engineering, Xi'an Aeronautical University, Xi'an, 710077 Shaanxi, China; ^2^Schools of Physics and Information Technology, Shaanxi Normal University, Xi'an, 710119 Shaanxi, China

## Abstract

To investigate the stability and dynamic characteristics of monolayer adsorbed on unsaturated lipid dioleoylphosphatidylcholine (DOPC) with varying concentrations of myelin basic protein (MBP), the system is studied by applying Langmuir technique and making atomic force microscope (AFM) observation, which is based on the mass conservation equation analysis method referred to in the thermodynamics theory. As indicated by surface pressure-mean molecular area (*π* − *A*) and surface pressure-adsorption time (*π* − *T*) isotherms, the physical properties of monolayer derived from the interaction of varying concentrations of MBP with liquid crystalline unsaturated lipid DOPC molecules were qualitatively studied. As revealed by surface morphology analysis with AFM, the micro region was expanded as the concentration of MBP in the subphase was on the increase, suggesting that hydrophobic interactions led to the MBP insertion, thus causing accumulation of the MBP on the surface of the monolayer. Experimental results have demonstrated that the partition coefficient of the interaction between MBP and unsaturated phospholipid DOPC and the molecular area of MBP adsorbed on the monolayer film was calculated using the mass conservation equation. In addition, not only does the varying concentration of MBP in the subphase exerts significant effects on the arrangement and conformation of DOPC monolayer, it also has certain guiding significance to exploring the structural changes to biofilm supramolecular aggregates as well as the pathogenesis and treatment of related diseases.

## 1. Introduction

**Figure 1:**
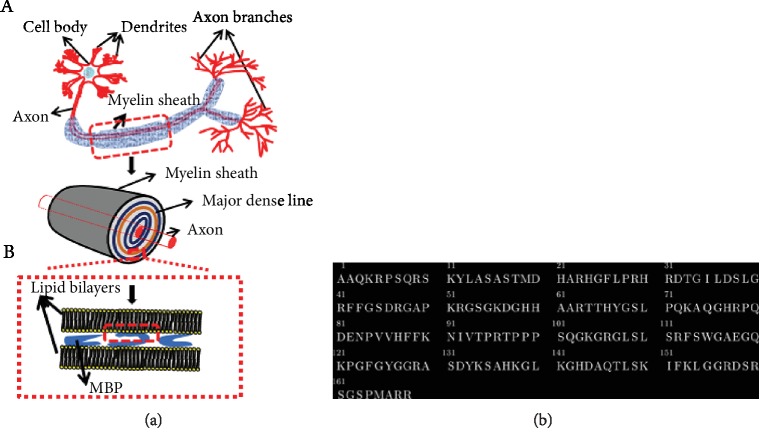
Schematic representation of the ((a), A) nervous system, ((a), B) myelin sheath, and (b) the amino acid sequence of 18.5 kDa MBP [7].

The myelin is an asymmetric multilamellar membrane structure derived from oligodendrocytes present in the central nervous system (CNS). As indicated by the results of biochemical molecular test, the structure of myelin sheath membrane is primarily a compact stack of lipid bilayers (mainly phospholipids), sphingolipid (mainly galactose brain glycosides), and proteins, as shown in Figure 1 [1–4].

**Figure 2:**
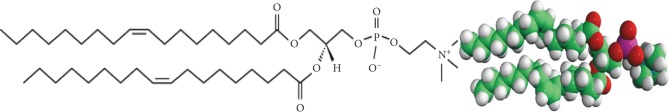
Chemical structures of the DOPC.

Although the interaction of MBP with different lipids has long been studied, such as the acidic lipids and neutral lipids, the quantitative study on the interaction between unsaturated lipid dioleoylphosphatidylcholine (DOPC) and myelin basic protein (MBP) remains excluded from discussion. DOPC is known as a relatively abundant glycerol phospholipid that exists in the myelin sheath, and the DOPC tail chain is in an unsaturated state with a low-phase transition temperature. The molecular structure is illustrated in Figure 2.

MBP is among the major proteins contained in the CNS and is an intrinsically disordered peripheral membrane protein [5]. The amino acid sequence of 18.5 kDa MBP is shown in Figure 1(b). Specifically, three amphipathic *α*-helices are present in proteins that bind to the myelin sheath, which are located at the N- and C-termini and in the central region. The 18.5 kDa splice form of MBP represents the predominant structure of myelin in the bovine and adult human brain [6, 7]. MBP promotes the formation of the main dense line of the myelin membrane, which maintains compactness of myelin sheath [8, 9]. In a neutral solution (at pH = 7), MBP shows a net positive charge of 19 and a isoelectric point (PI) of 10.6 [10–12]. In aqueous solution, isolated MBP barely has any regular structure. Nevertheless, the binding with lipids causes it to form ordered secondary structure and undergo surface conformational changes [13–15].

As demonstrated by the recent studies, taking measurement of the maximum insertion pressure and surface pressure of MBP for different lipid monolayers could provide information about the interaction [16]. Besides, the development of an amorphous protein phase of MBP between two membrane bilayers has been observed in other studies [17]. In addition, plenty of qualitative researches have been conducted into the interaction of MBP with saturated phospholipid via electrostatic and hydrophobic interactions [18–20]. Therefore, the study on the interaction between MBP and unsaturated lipid DOPC molecules is believed to have substantial medical value and biological significance to judging the pathogenesis and determining the treatment of central nervous system diseases.

Furthermore, we have followed up on our previous work [11, 21, 22] and performed a AFM study on the effects created by MBP adsorption on DOPC monolayer. Therefore, the aim of this work is to apply the Langmuir-Blodgett (LB) monolayer model and AFM to figure out the physical properties displayed by bionic membranes derived from the molecular interaction with varying concentrations of MBP and unsaturated lipid DOPC molecules. LB technology is applied to transfer insoluble monolayer suspended on gas-liquid interface to solid substrate through membrane balance [23]. Our results revealed the quantitative relationship between MBP and unsaturated lipid DOPC molecules and the adsorption kinetics of protein molecules onto DOPC monolayers were quantitatively analyzed using thermodynamic method. In addition, the aggregation state and molecular arrangement of MBP molecules adsorbed on DOPC monolayers were observed with the assistance of AFM. Besides, we gained insights into the thermodynamic and topographic behavior of this complex mixture.

## 2. Materials and Methods

### 2.1. Materials

Following the process established by Deibler et al., MBP was purchased from Merck Millipore Chemical Reagent Company, purified and isolated from bovine brain, prior to being dialyzed against pure water [24, 25]. The MBP was diluted in a solution of Tris-HCl (10 mM, pH = 7.2), and finally the concentration required for the experiment was 1.0 × 10^−9^~3.8 × 10^−9^ M. Trimethylol methylamine (tris (hydroxymethyl) aminometha, purity ≥ 99%). Dioleoylphosphatidylcholine (DOPC) was sourced from Sigma Chemical Reagents and can be used straightaway without the need for further purification. In the study of the monolayer, DOPC was dissolved in a mixture of chloroform/methanol (3 : 1, *v*/*v*), and the storage solution with the concentration of 1 mg/mL was finally derived. The experimental water was obtained from a three-distilled water system, with a resistivity of 18.2 M*Ω*cm.

### 2.2. Determination of Surface Pressure-Mean Molecular Area (*π* − *A*) Isotherm

The *π* − *A* isotherms of the phosphatide monolayer on the air/subphase interface were measured by using KSV-Minitrough (KSV, Finland) Langmuir film balance. The platinum Wilhelmy plate on the instrument can be taken as a pressure sensor. The measured surface pressure ranges from 0 to 150 mN/m, and the accuracy is 0.01 mN/m. The subphase used to generate the monolayers is the Tris-HCl solution with pH of 7.2 and concentration of 10 mM. A microsyringe was used to add a certain volume of lipid solution to the pure water subphase or to the subphase containing certain concentrations of MBP. Subsequently, the solvent was volatilized for 15 minutes, and the monolayer was compressed at the speed of 10 mm/min and monitored by a pressure sensor in real time. The relationship curve between the surface pressure and the mean molecular area is plotted. In addition, the monolayer film is capable to be compressed to the required film pressure, the barrier is stopped, the area of the monolayer remains unchanged, and the curve of the *π* varying with *T* can be recorded. Finally, the changes to surface pressure and time of lipid monolayers were captured.

The surface pressure of the monolayer prepared in this paper is 10 mN/m. The monolayer is transferred to the surface of the newly dissociated mica substrate using the vertical Czochralski transfer method (the transfer ratio is 1).

In the above experiments, the subphase temperature was kept at 20 ± 1°C by external constant temperature water cycle. Before each experiment was conducted, the trough and barrier were repeatedly cleaned with anhydrous ethanol, before being rinsed with ultrapure water. All the experiments were repeated three times to ensure the repeatability of the curve.

### 2.3. Atomic Force Microscope Observation

The monolayers transferred to the mica substrate were naturally dried at room temperature and then carried out with a commercial AFM (SPM-9500-J3 Shimadzu Instruments Co. Ltd., Kyoto, Japan). The microcantilever and probe represent the major components involved in the image acquisition of AFM. The AFM probe used was a micro V-shaped cantilever (Olympus Optical Co. Ltd., Kyoto, Japan). The probe material was Si_3_N_4_ and the elastic constant was 0.06 N/m. The whole detection process was carried out at room temperature, with a scanning rate of 1 Hz and a pixel point of 512 × 512.

### 2.4. Thermodynamic Theoretical Analysis of MBP/DOPC Interaction

In our isothermic experiments, the phospholipid monolayers were spread across the surface of subphase with a certain concentration of MBP. Based on the prior studies, phospholipids are considered as an insoluble surfactant that is only capable to be located at the air/subphase interface, which makes the concentration of phospholipids negligible. MBP is known as a soluble surfactant that has the capability to distinguish between interface and subphase, for which it can interact with phospholipids at the air/subphase interface, and the concentration of MBP in the subphase cannot be discounted [26]. The temperature of the entire process system remains unchanged. The total molar number of phospholipid and protein in the system, the surface area, and the surface pressure of the monolayer can be measured. These parameters can be used to calculate not only the partition ratio of MBP in monolayer interface and subphase, respectively, but also the membrane area occupied by protein molecules. Based on the research carried out in Schwarz and Taylor [27, 28], the law of conservation of mass can be expressed as follows:

(1) $$\begin{array}{c} n_{p}^{0}=n_{l}r+n_{p}^{s}, \end{array}$$

where *r* = *n*_*p*_/*n*_*l*_, *n*_*p*_^0^ represents the total amount of protein added, *n*_*l*_ indicates the total amount of lipids in the system, *r* denotes the mixing coefficient, *n*_*p*_^*s*^ is referred to as the amount of protein in the subphase, and *n*_*p*_ stands for the amount of interfacial domain protein. In order to facilitate the research, all quantities are expressed as amount of substance.

According to Schwarz and Taylor [27, 28], when the concentration of protein in the subphase is so low that its nonideal interaction can be ignored, the chemical potential change in water can be made negligible, that is to say, under the equilibrium condition, *r* and *n*_*p*_^*s*^ are determined solely by the increase in the area per mole of lipid (Δ*A*^∗^) and surface pressure (*π*), where Δ*A*^∗^ can be expressed by the amount of increase in lipid area (Δ*A*) and the amount of total lipid (*n*_*l*_), namely, Δ*A*∗ = Δ*A*/*n*_*l*_. When the surface pressure and the increase per mole of lipid area remain unchanged, *r* and *n*_*p*_^*s*^ are constant. Therefore, according to the mass conservation plots, *n*_*l*_ is a linear function of *n*_*p*_^0^. All parameters can be analyzed with the experimental data.

In order to obtain the area occupied by MBP molecules, it is necessary to analyze the increase in the surface area following protein adsorption onto lipid monolayers from the experiment. The amount of increase per mole of lipid area (Δ*A*^∗^) is analyzed as a function of surface pressure (*π*) by comparing the presence or absence of protein in the subphase. The increase in area is attributed to not only the surface area of the adsorbed protein (*rA*_*p*_) but also the change to the area of each lipid molecule generated by lipid-protein interaction (Δ*A*^∗^).

(2) $$\begin{array}{c} \Delta A^{*}=\Delta A_{l}+{rA}_{p}. \end{array}$$

As the area occupied by each lipid molecule is not fixed when the protein is inserted into the lipid monolayer, the surface area of the protein adsorbed on the interface of the monolayer is noticeably larger than the area occupied by each lipid molecule. Therefore, according to the following equation, the molecular area of the protein can be calculated as follows:

(3) $$\begin{array}{c} A_{p}\left(\pi \right)=\Delta A^{*}\left(\pi \right)/r. \end{array}$$

## 3. Results and Discussions

### 3.1. Theoretical Analysis of Interaction between Different Concentrations of MBP and DOPC Monolayers at Air/Subphase Interface

Distinct from other small molecules, surfactants show poor solubility in water. It is thus highly challenging to determine the solubility of surfactants in water by thermodynamics or other conventional methods, such as adsorption filtration combined with a detector. Therefore, a certain amount of surfactant DOPC solution is added to the subphase with varying concentrations of MBP. Ideally, when the monolayer membrane is compressed, the phospholipid molecules are consistently at the interface, and the number of phospholipid molecules entering into the subphase by desorption is very low. It can be considered that there is no loss in the process of compressing the monolayers. Therefore, when the temperature is unchanged, the surface pressure of monolayer on the air/subphase interface is monitored by using the traditional Langmuir film balance. For a given set of isotherms (*π* − *A*) with varying amounts of lipid and protein, the mass conservation equation given by equation (1) was applied to calculate not only the partition ratio of MBP at the interface of the monolayer and the subphase but also the membrane area occupied by the protein molecules.

**Figure 3:**
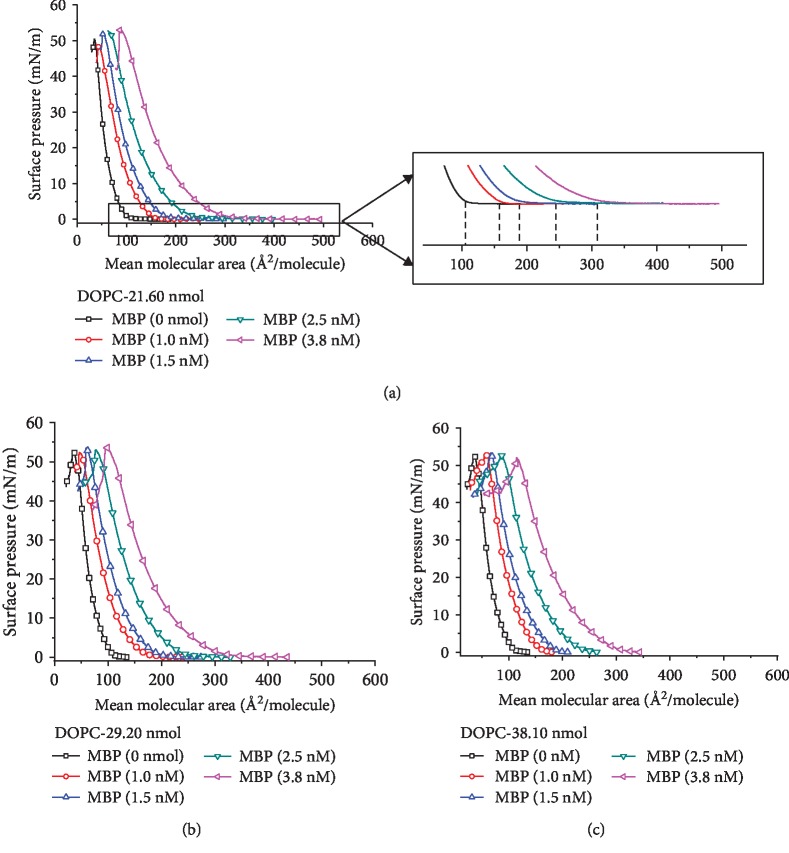
Effect of different concentrations of MBP in subphase on DOPC monolayer. The amount of the lipid is 21.6 nmol (a), 29.2 nmol (b), and 38.1 nmol (c).

To clarify the interaction of MBP adsorbed from the subphase with the monolayer of unsaturated lipid DOPC, *π* − *A* measurements were performed.  Figure 3 shows *π* − *A* isotherms of DOPC monolayers on the buffer subphase containing MBP at varying concentrations between 1.0 and 3.8 nM. The pure monolayer of DOPC exhibits typical isotherm with a change in slope at *π* ≈ 21 mN/m and area of 73 Å^2^ molecule, where a phase transition from the liquid state condensed to the solid state occurs [29]. In addition, as illustrated in Figure 3, following an increase in MBP concentration in the subphase, the isotherm of all the DOPC studied were shifted to higher molecular areas, indicating that the monolayers underwent expansion. It can be seen from the enlarged view of Figure 3(a) that the initial area of the pure DOPC monolayer is about 105 Å^2^. Besides, when the concentration of MBP in the subphase is 1.0, 1.5, 2.5, and 3.8 nM, the initial area is about 155, 190, 245, and 307 Å^2^, respectively. As implied by the experimental result, MBP adsorbed on the interface of DOPC monolayers interacts with it and expands the molecular area.

**Figure 4:**
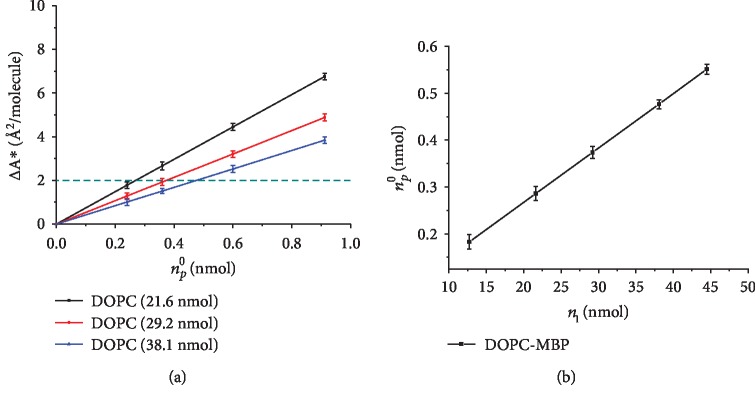
Mass conservation plots for DOPC according to Eq. (1) at given surface pressures of 10 mN/m, functional relationships between Δ*A*^∗^ − *n*_*p*_^0^ (a) and *n*_*l*_ − *n*_*p*_^0^ (b).

For penetrating investigation into the combination of MBP and POPC molecules with varying concentrations in the subphase, based on the mass conservation equation, *n*_*p*_^0^ = *n*_*l*_*r* + *n*_*p*_^*s*^, the functional relationship between Δ*A*^∗^ and *n*_*p*_^0^ under fixed surface pressure (10 mN/m) can be obtained from Figure 3. The more the total amount of lipid added to the interface, the smaller the linear slope. When Δ*A*^∗^ remains unchanged, the functional relationship between *n*_*l*_ and *n*_*p*_^0^ can be determined. As shown in Figure 4, the slope represents the distribution coefficient of the interaction between MBP and POPC. The *y*-axis intercept refers to the amount of MBP in the subphase. As a result, when Δ*A*^∗^ = 2, the partition coefficient *r* = 0.012, the amount of MBP in the subphase is 0.02 (*n*_*p*_^*s*^ = 0.02), and it can be known by calculation that one MBP molecule is capable to bind 76 ± 3 DOPC molecules. In addition, according to the equation *Α*_*p*_(*π*) = Δ*A*^∗^(*π*)/*r*, the molecular area of MBP at varying concentrations can be ascertained. When the surface pressure is 10 mN/m, the amount of DOPC added to the interface is 29.20 nmol, and the concentration of MBP is 1.0 nM, the molecular area of MBP is 107.05 Å^2^. When the concentration reaches 1.5, 2.5, and 3.8 nM, the molecular area of MBP is 160.39, 267.35, and 406.42 Å^2^, respectively. When the amount of DOPC added to the interface is 21.60 and 38.10 nmol, the molecular area of varying concentrations of MBP in the subphase is indicated in Table 1. It can be established from theoretical calculation that the molecular area occupied by one protein is substantially larger than the molecular area occupied by one lipid molecule.

### 3.2. Adsorption of MBP on DOPC Monolayers

**Figure 5:**
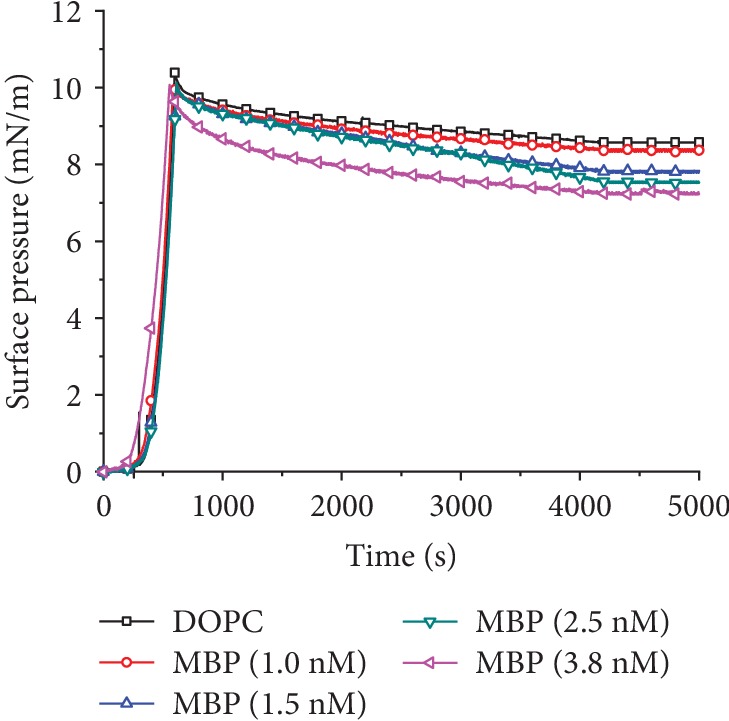
When the surface pressure is 10 mN/m, MBP adsorbs to the DOPC monolayer surface pressure curve with time. The concentration of MBP in subphase is 0, 1.0, 1.5, 2.5, and 3.8 nM, respectively.

The interfacial adsorption of DOPC systems has been evaluated in the absence or presence of increasing concentrations of MBP. As revealed by Figure 5, when the initial surface pressure reaches 10 mN/m, the surface pressure tends to show a declining trend as a whole, suggesting that the lipid molecules at the interface after the compression stop will rearrange or the MBP molecules with high relative molecular weight will carry some DOPC molecules into the subphase, thus causing the surface pressure to drop. From the *π* − *T* curve, it can be visually analyzed that as the concentration of MBP is on the increase in the subphase, the kinetics and extent of surface pressure changes are affected significantly by the lipid compositions of the monolayers. The declining amount from small to large is as follows: Δ*π*_0nM_ < Δ*π*_1.0nM_ < Δ*π*_1.5nM_ < Δ*π*_2.5nM_ < Δ*π*_3.8nM_. It was discovered that a higher concentration of MBP in the subphase led to a stronger interaction between MBP and DOPC molecules. Moreover, it was found that more MBP molecules transferred more DOPC molecules to the subphase and interacted with them, thus making the monolayer sparser. The surface pressure failed to further decline beyond 3500 s and ends up reaching the equilibrium state, which indicates either that MBP has been adsorbed on the DOPC monolayer or that MBP has entered into the subphase to give rise to the aggregate structure. Therefore, the interactions between the lipid monolayers and MBP are believed as hydrophobic interactions. In conclusion, the adsorption capacity of MBP on DOPC monolayer is largely reliant on the concentration of MBP in the subphase and the lipid composition of monolayers on the air/subphase interface.

### 3.3. AFM Observation of Different Concentrations of MBP Adsorbed on Different Lipid Membranes

**Figure 6:**
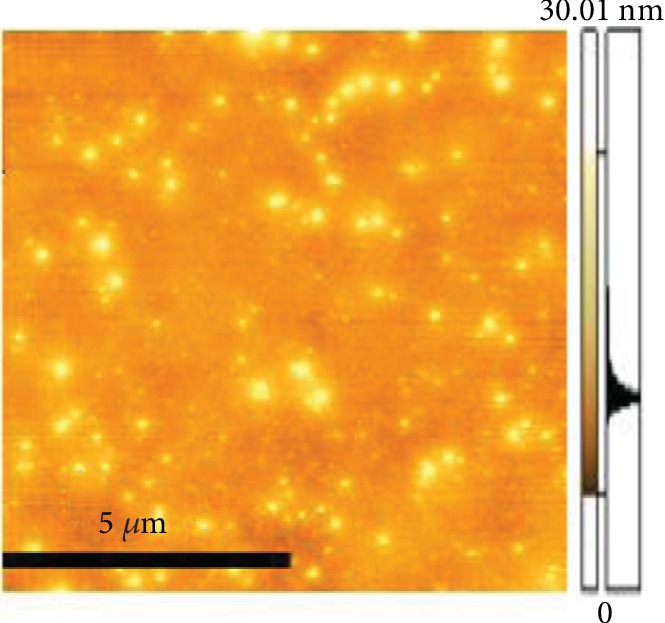
AFM image of MBP molecular deposited at 10 mN/m on mica by Langmuir-Blodgett. Scale bar: 5 *μ*m.

**Figure 7:**
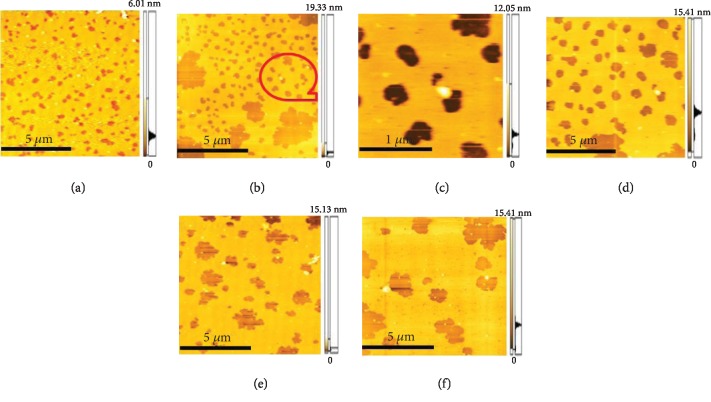
AFM image (10 *μ*m × 10 *μ*m) of the binary MBP/DOPC monolayers at different concentrations of MBP. The concentration of MBP in subphase is 0 nM (a), 1.0 nM (b), 1.5 nM (d), 2.5 nM (e), and 3.8 nM (f), respectively, where (c) is the magnified picture of (b). The scale bars are shown for each image.

**Figure 8:**
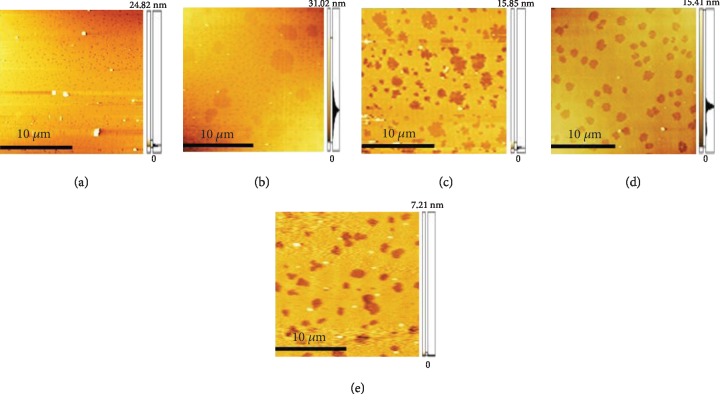
AFM image (20 *μ*m × 20 *μ*m) of the binary MBP/DOPC monolayers at different concentrations of MBP. The concentration of MBP in subphase is 0 nM (a), 1.0 nM (b), 1.5 nM (c), 2.5 nM (d), and 3.8 nM (e), respectively. Scale bars are 10 *μ*m in each image.

**Figure 9:**
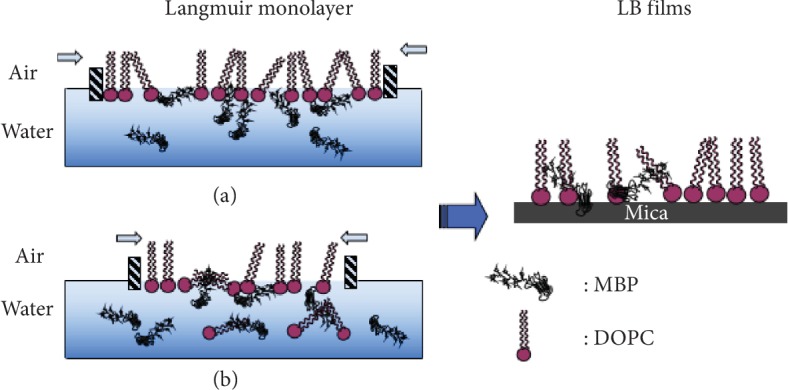
Kinetic model of interaction between MBP and unsaturated lipid DOPC.

In this system, when the surface pressure was 10 mN/m, the monolayer of the air/subphase interface was transferred to the smooth mica substrate and observed by AFM. This system was purposed to determine whether MBP would cause the conformational change in DOPC monolayer. As clearly revealed by the AFM image, the protein molecules were dispersed across the surface of mica (Figure 6).  Figure 7 illustrates the surface topography of the mixed monolayers with varying concentrations of MBP and DOPC, and it can be found that a large number of protein particles are scattered across the surface microdomains of the mixed monolayers. The scale bars of each image is 5 *μ*m. As shown in Figure 7(a), the domains are homogeneously distributed on the mica substrate in the absence of MBP, the microdomains exhibit a uniform “petal-like” structure, and the molecular arrangement is relatively close. When the concentration of MBP in the subphase is 1.0 nM (Figure 7(b)), the surface morphology of the monolayer changes massively, and the “flower-shaped” structure of the microdomains is made more obvious.  Figure 7(c) clearly indicates the petal-like structure (Figure 7(c) is the magnified picture of Figure 7(b)). With a rising protein concentration in the subphase (Figure 7(d)), the petal-like structure of the microdomain grows incrementally. When the concentration of MBP is 2.5 nM, the petal-like structure of the monolayer continues to grow, and it is observed that sporadic proteins are scattered across the surface of the microdomain. When the concentration of MBP rises to 3.8 nM, the microstructural area of the monolayers is found to be larger and sparser. Overall, the greater the concentration of MBP in the subphase, the larger the area of petal-like microdomain and the lipid membrane structure is becoming increasingly sparse. In addition, it can be spotted that the protein particles are scattered across the surface of the monolayer. Meanwhile, as the concentration is on the rise, MBP will aggregate, which suggests that the impact of MBP molecule has been adsorbed by the DOPC domains on monolayer morphology that will have interaction with the DOPC molecule.  Figure 8 presents the characterization of the morphological changes to mixed monolayers in either the presence or absence of MBP, with the scale bars of each image being 10 *μ*m. The dynamic process of interaction between unsaturated lipid DOPC and MBP can be understood more intuitively using the model diagram shown in Figure 9. Firstly, the interaction between MBP and DOPC molecules causes MBP molecules to adsorb the interface (Figure 9(a)). Secondly, during the compression process, MBP molecules are squeezed out of the interface and carry some phospholipid molecules into the subphase, thus causing the monolayers to be further loosened (Figure 9(b)).

## 4. Conclusion

The research provides firm experimental evidence for the interaction between MBP and DOPC. In the present work, the surface activity of DOPC with the unsaturated hydrocarbon moiety has been thoroughly studied. The results obtained from the experimental demonstrate that (1) we have succeeded in applying mathematical and physical methods to determine a possible adsorption of MBP in the DOPC monolayer. These results have confirmed the interesting and novel structural and thermodynamic properties. Based on the law of mass conservation, it is calculated that one MBP molecule is capable to bind 76 ± 3 DOPC molecules and the molecular area occupied by MBP at varying concentrations. (2) Considering the *π* − *T* isotherms, it can be judged that MBP penetrates into the DOPC monolayer in the liquid-expanded phase and it is squeezed out of the monolayer in the liquid-condensed phase. Furthermore, when surface pressure is low, MBP can be adsorbed into the lipid monolayer, thus increasing the lipid area per molecule. (3) The AFM image further indicates that MBP has close interaction with DOPC, which is likely to induce conformational change in DOPC monolayer. In summary, the application of thermodynamic properties to research the interaction between MBP and DOPC molecules provides a better theoretical and experimental basis to gain an in-depth understanding of MBP to maintain myelin stability and tightness.

## Figures and Tables

**Table 1:** The molecular area occupied by MBP in the subphase when the air/subphase interface is added with different amounts of lipid.

Amount of lipid (*n*_*l*_/nmol)	Molecular area occupied by MBP (*Α*_*p*_(*π*) = *Å*^2^)			
	1.0 nM	1.5 nM	2.5 nM	3.8 nM
21.6	106.56	175.92	316.94	498.69
29.2	107.05	160.39	267.35	406.42
38.1	84.27	125.35	209.07	318.31

## Data Availability

The data used to support the findings of this study are available from the corresponding author upon request.
